# Increased *Trypanosoma brucei* cathepsin-L activity inhibits human serum-mediated trypanolysis

**DOI:** 10.15698/mic2014.08.162

**Published:** 2014-07-14

**Authors:** Sam Alsford

**Affiliations:** 1 London School of Hygiene & Tropical Medicine, Keppel Street, London WC1E 7HT, UK.

**Keywords:** Trypanosoma brucei, trypanolysis, cathepsin-L, innate immunity, lysosome

## Abstract

Most African trypanosomes, including the veterinary species *Trypanosoma brucei brucei *and *T. congolense *are susceptible to lysis by human serum. A recent study by Alsford *et al.* [PLoS Pathogens (2014) 10, e1004130] has identified a *T. b. brucei *lysosomal cathepsin with an inhibitory effect on human serum’s trypanolytic action.

African trypanosomes are a group of flagellated protozoan parasites endemic to sub-Saharan Africa. They are mostly non-infectious to humans, with the exception of two subspecies, *T. b. gambiense *and *T. b. rhodesiense*, the causative agents of human African trypanosomiasis (HAT; also known as sleeping sickness) in west and central Africa and east Africa, respectively, where up to 70 million people are regarded as at risk. African trypanosomes, including *T. b. brucei *and *T. congolense*, also cause nagana, a wasting disease in livestock, which is thought to have stifled agricultural development in the region over millennia. *T. brucei *is renowned for its ability to evade the adaptive immune system of its mammalian host by repeatedly replacing its variant surface glycoprotein (VSG) coat, a process known as antigenic variation. The human-infective subspecies have also evolved distinct mechanisms to evade human innate defences that normally cause rapid lysis of invading veterinary trypanosomes.

Over recent years, the work of several groups, including those of Etienne Pays (Free University of Brussels, Belgium), Stephen Hajduk (University of Georgia, USA), Jayne Raper (City University New York, USA) and Annette MacLeod (University of Glasgow, UK), has advanced our understanding of the mechanisms underlying human serum-mediated trypanolysis, and the factors that render *T. b. gambiense *and *T. b. rhodesiense *resistant to this attack. Human serum contains two trypanolytic factors, TLF1 (a constituent of high density lipoprotein) and TLF2 (an IgM/apolipoprotein-A1 complex), both of which contain apolipoprotein-L1 (APOL1), the trypanolytic component. *T. brucei *takes up the TLFs via receptor-mediated endocytosis. In the case of TLF1, this is dependent on the interaction between haptoglobin-related protein, found in the complex, and the trypanosome haptoglobin-haemoglobin receptor (HpHbR); it is currently unknown how TLF2 enters the parasite. Transit through the endocytic network brings TLF-APOL1 to the lysosome where the acidic pH results in a conformational change in APOL1, allowing it to form pores in the lysosomal membrane, leading to osmotic swelling and parasite lysis. *T. b. gambiense *and *T. b. rhodesiense *have evolved distinct VSG-based mechanisms to circumvent this lytic attack. In addition to their surface coat VSG, both express subspecies-specific truncated VSGs that are retained in the endocytic system: *T. b. gambiense*-specific glycoprotein (TgsGP) and serum-resistance associated (SRA) protein, respectively. SRA interacts directly with APOL1, blocking its access to the lysosomal membrane. In contrast, human serum resistance in *T. b. gambiense *relies on both gain-of-function and loss-of-function mutations. TgsGP is thought to stiffen the membranes of the endocytic network, rendering them less susceptible to attack by APOL1, but resistance also depends on reduced TLF1 uptake by TbHpHbR, due to a single amino acid substitution.

While it’s known how TLF1 enters African trypanosomes, how APOL1 kills the parasite, and how the human-infective subspecies are able to resist human serum lysis, there are still significant gaps in our understanding of this process. For example, what components of the endocytic system are responsible for transiting TLF to the lysosome, and how does TLF2 enter the parasite? Other than HpHbR, what else makes *T. b. brucei *susceptible to lysis by human serum, and could changes in these factors represent alternative routes to resistance? The recent development of a bloodstream-form *T. b. brucei *RNA interference library enabled the identification of a complex network of proteins required for the receptor-mediated endocytosis of the anti-HAT drug, suramin. Selection of the RNAi library with human serum was expected to identify a similarly complex set of proteins responsible for transit of the massive (>0.5 MDa) TLF complexes through the parasite’s endocytic system to the lysosome. Perhaps surprisingly, only four proteins were identified in the screen, including the previously characterised HpHbR and a lysosomal membrane protein, p67, already known to influence sensitivity to human serum, as well as inhibitor of cysteine peptidase (ICP) and a putative channel protein. Only one of these proteins, p67, promotes both suramin efficacy and human serum sensitivity, highlighting the importance of the lysosome to the action of both toxins, but also revealing that their successful transit through the parasite’s endocytic system and efficacy are dependent on different cohorts of proteins.

The identification of ICP highlighted the potential role of trypanosome cysteine peptidases in resisting trypanolysis by human serum. Targeted RNAi knockdown of the two *T. b. brucei *cathepsins, CATB and CATL, in *icp *null parasites revealed that CATL, but not CATB, could influence human serum sensitivity, though only in the absence of ICP. Intriguingly, in the presence of ICP, knockdown of CATL had no effect on parasite sensitivity to human serum, indicating that ICP’s repressive action is modulated in response to CATL activity. At present, how CATL influences trypanolysis is unclear; there are several possibilities (Figure 1). As in other eukaryotes, CATL localises to the lysosome in *T. b. brucei*, where the low pH promotes its activity. The peptidase may target TLF-APOL1, free APOL1, or both, once they reach the lysosome, though the relative sensitivity of APOL1 and the TLFs to CATL-mediated proteolysis is unknown. One possibility is that being embedded within a TLF complex affords APOL1 some protection from attack. It has been proposed that the TLF particle interacts with the lysosomal membrane, enabling the membrane integration of the constituent APOL1, and thereby limiting the amount of free APOL1 in the lysosome. In this situation, additional CATL activity may lead to TLF disruption and release of APOL1, exposing it to proteolysis by CATL or other lysosomal factors. It should be noted, however, that even in the absence of ICP, and in the presence of presumably fully active CATL, *T. b. brucei *is still highly sensitive to human serum, with an EC50 of ~0.002% compared to ~0.00025% for wild-type parasites. Clearly, release of CATL from the inhibitory influence of ICP by itself is insufficient to fully block APOL1’s assault on the parasite’s lysosomal membrane. However, in a parasite like *T. b. gambiense*, where TLF1 uptake is already significantly reduced and lysosomal membranes may be less susceptible due to the action of TgsGP, such a relatively small drop in sensitivity may be all that’s required to make the final step to being fully resistant to human serum.

**Figure 1:**
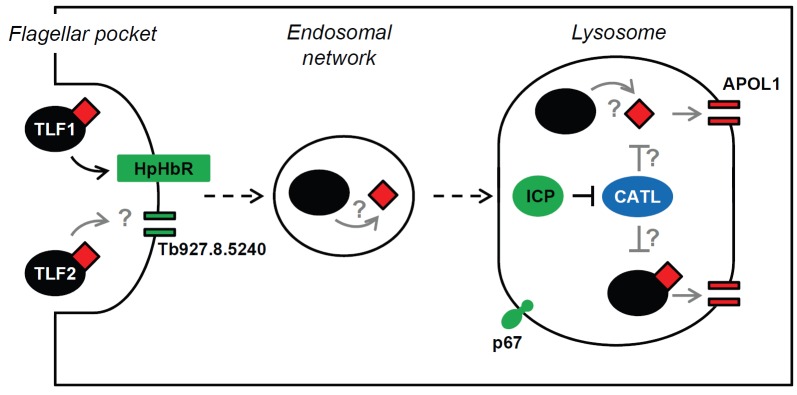
FIGURE 1: Speculations on the interactions between TLF, APOL1 and CATL, and a possible role for the putative channel protein, Tb927.8.5240. TLF1 is known to enter *T. b. brucei *via HpHbR at the flagellar pocket membrane, and then transit to the lysosome where the lytic component, APOL1, forms pores in the lysosomal membrane leading to osmotic swelling and parasite lysis. The mechanism for TLF2 entry is unknown, though it has been speculated that it may interact with surface VSG, which is rapidly endocytosed. The putative channel protein, Tb927.8.5240, represents a possible alternative uptake mechanism; however, the localisation detailed above is speculative. It is unknown whether or where within the endocytic network APOL1 dissociates from TLF. CATL is presumed to act upon either the lytic complexes or APOL1 in the lysosome, as its activity is dependent upon the acidic pH found in this organelle. Reduced human serum sensitivity is due to ‘loss-of-function’ (Green) or ‘gain-of-function’ (Blue); the latter includes SRA and TgsGP, which have been omitted for clarity (see main text for details).

The identification of only four proteins that make a significant contribution to the human serum sensitivity of *T. b. brucei*, suggests that the routes to human serum resistance available to this parasite are very limited. However, it seems unlikely that human serum sensitivity in *T. b. brucei *is influenced only by these four proteins. For example, there is likely a network of proteins involved in determining lysosomal pH, a property that is critical for the action of APOL1. Although many of these proteins may be essential for parasite survival, at least representatives of this group would have been expected to have emerged following selection of the bloodstream form *T. b. brucei *RNAi library in human serum. Indeed, the presence of short RNAi fragments in the library, which typically result in limited knockdown, has allowed the contribution to toxin efficacy of otherwise essential proteins to be identified in previous screens. Following selection of the RNAi library in human serum, the outputs were dominated by HpHbR (representing more than 73% of all mapped reads), the loss of which is known to render *T. b. brucei *highly resistant to trypanolysis. Therefore, factors whose depletion only provides a marginal advantage to treated parasites, but which still make a significant contribution to TLF-APOL1 activity, may be out-competed by those depleted for HpHbR. Selecting the bloodstream-form *T. b. brucei *RNAi library with purified TLF2 or recombinant human APOL1, thereby preventing the selection of parasites depleted for HpHbR, should enable the identification of parasite factors responsible for TLF2 uptake and lysosomal pH regulation, respectively. It will be intriguing to see what more these screens reveal about the role of *T. b. brucei *proteins in the promotion of human serum trypanolysis.

